# Diagnostic value of dual-energy CT and clinicopathological and imaging feature analysis of mixed endometrial stromal and smooth muscle tumors with intracardiac extension

**DOI:** 10.3389/fcvm.2022.917399

**Published:** 2022-09-15

**Authors:** Yi-yang Liu, Zhan Yu, Rui Wang, Kui-sheng Chen, Song-wei Yue, Jun Li, Xue-mei Gao, Chang-mao Ding, Zi-xin Wu, Xi Zhao, Jian-bo Gao

**Affiliations:** ^1^Department of Radiology, The First Affiliated Hospital of Zhengzhou University, Zhengzhou, China; ^2^Henan Key Laboratory of Imaging Diagnosis and Treatment for Digestive System Tumor, Zhengzhou, China; ^3^Department of Pathology, The First Affiliated Hospital of Zhengzhou University, Zhengzhou, China; ^4^Department of Magnetic Resonance, The First Affiliated Hospital of Zhengzhou University, Zhengzhou, China; ^5^Department of Urology Surgery, The First Affiliated Hospital of Zhengzhou University, Zhengzhou, China; ^6^Department of Customer Services, Siemens Healthineers, Shanghai, China

**Keywords:** intracardiac extension, MESSMT, intravascular extension, dual-energy CT, diagnosis

## Abstract

**Objective:**

To describe the clinicopathological and imaging features of mixed endometrial stromal and smooth muscle tumors with intracardiac extension and to explore the diagnostic value of dual-energy computed tomography (DECT) for this rare entity.

**Materials and methods:**

This retrospective study analyzed the clinicopathological data and images of a 41-year-old female patient with pathologically documented mixed endometrial stromal and smooth muscle tumors with intracardiac extension who had undergone DECT examination. Seven virtual monoenergetic images (VMIs) in 10-kiloelectron volt (keV) intervals (range = 40–100 keV), iodine density (ID) maps, and Z effective (Zeff) maps were reconstructed, and lesion conspicuity was assessed. Tumor homology was analyzed using quantitative DECT parameters and energy spectrum attenuation curve.

**Results:**

The patient complained of a 10-day history of bilateral lower extremity edema. Computed tomography showed a hypoattenuating filling defect located within the paracervical vein that extended into the right atrium to the ventricle through the right iliac veins and inferior vena cava (IVC). Intracardiac and intravenous lesions mainly demonstrated moderate progressive enhancement, with localized non-enhancing necrotic areas on contrast-enhanced CT. Multiple nodules showing progressive enhancement (long-T1 signal, long-T2 signal) were observed at the fundus of the uterus on dynamic contrast-enhanced magnetic resonance imaging (MRI), which were deemed the primary lesions of the tumor. Overall, the tumor was characterized by a small primary lesion with extensive vascular extension. In addition, the 40 keV VMIs reconstructions were found to provide best visualization for the early detection of tumors.

**Conclusion:**

Although a definitive diagnosis of MESSMT with intracardiac extension requires confirmation by histopathological examination, imaging examination can be used to characterize the extent of the lesion. The dual-energy dataset facilitates tumor visualization and homology evaluation.

## Introduction

Mixed endometrial stromal and smooth muscle tumor (MESSMT) is an uncommon mesenchymal uterine tumor characterized by the presence of both endometrial stromal and smooth muscle tissue (1). Such tumors with intracardiac extension are extremely rare. A literature search revealed only five cases of MESSMT with intracardiac extension in the English medical literature (2–6). MESSMT can be misdiagnosed and is easily missed; when combined with intracardiac extension, the condition is even more complicated. Thus, accurate preoperative diagnosis is helpful for guiding the choice of treatment and predicting the prognosis of these patients (5). Imaging examination is an important tool for the diagnosis of uterine lesions and can be used to determine whether the lesion has invaded other organs. However, to the best of our knowledge, the imaging appearance of MESSMT with intracardiac extension has not been systematically summarized due to its low incidence rate. Herein, we describe the radiological appearance and clinical-pathologic features of a rare case of MESSMT with intracardiac extension and review the related literature. In addition, we explore the added value of dual-energy datasets for this complex entity.

## Materials and methods

### Study cases

This retrospective study was approved by an ethical committee, and the requirement for informed consent was waived.

The clinicopathological features and imaging findings (CT, MRI, and US) of a case of mixed endometrial stromal and smooth muscle tumor with intracardiac extension in a 40-year-old woman are analyzed. We searched the literature using medical terms (Mixed endometrial stromal and smooth muscle tumor [MeSH] AND Intracardiac extension [MeSH] OR Heart metastasis [MeSH]) in PubMed; previously reported cases are summarized and explored (Table 1).

**TABLE 1:** Clinical features of previously reported and current study cases of mixed endometrial stromal and smooth muscle tumor (MESSMT) with intracardiac extension.

Case	Age	Complaint	Tumor marker	Original diagnosis	Extended range	Gross features of uterine lesions	Surgery	Prognosis
(1) Whitlatch and Meyer (2)	50	Progressive dyspnea	*NE*	IVL	IVC, right atrium	Mass	Staging surgery	8 y. Die
(2) Mikami et al. (3)	24	Pelvic masses	*NE*	IVL	IVC, right atrium, right lung	Mass	Staging surgery	12 y. No recurrence
(3) Coganow et al. (4)	47	Dyspnea on exertion, left-sided chest discomfort, and lower extremity edema	*NE*	*NE*	IVC, right atrial pulmonary	*NE*	Single-Stage operation	9 mo. No recurrence
(4) Zhang et al. (5)	45	Dyspnea on exertion	*NE*	IVL	IVC, right atrial right ventricle	Mass	Staging surgery	3 mo. No recurrence
(5) Huang et al. (6)	45	Chest tightness after activity and mild lower extremity edema	CA125↑	*NE*	IVC, right atrial right ventricle	Mass	Staging surgery	5 mo. No recurrence
(6) Present Case	40	Lower extremity edema	TAP↑	IVL	IVC, right atrial right ventricle	Multiple nodules and dilated vascular structures	Single-Stage operation	8 y. No recurrence

NE, no evaluation; IVC, inferior vena cava; IVL, intravenous leiomyomatosis; y, year; mo, month.

### Subjective and objective dual-energy computed tomography image analysis

The virtual monochromatic images (VMIs) (40–100 keV, at an interval of 10 keV), iodine density (ID) maps, and Z effective (Zeff) maps were reconstructed from the dual-energy dataset using dedicated workstations (Syngo.via, Version VB20, Siemens Healthineers). Notably, 70 keV VMI is equivalent to 120 kV conventional single energy CT acquisition (7).

To explore the visualization effect of early vascular invasion in MESSMT with the reconstructed dual-energy images, we evaluated the lesion conspicuity of the involved paracervical vein by employing a five-point Likert scale (1: barely perceived; 2: subtly visualized; 3: fairly detectable 4: definitely detected; 5: strikingly evident/easily spotted) (8).

Furthermore, VMI has been confirmed to improve the signal-to-noise ratio (SNR) and the contrast-to-noise ratio (CNR) in tumor imaging (9–11). To determine the optimal energy levels of VMI reconstruction for objective assessment of early vascular invasion, VMIs were measured and compared. Circular regions of interest (ROIs) with constant size of 1 cm^2^ were placed on the involved paracervical vein, gluteal muscle, and subcutaneous fat of hip across three adjacent levels, avoiding large vessels and any area with obviously gross necrosis, or calcification. The mean CT attenuation values and standard deviation (SD) in VMIs were obtained within the ROIs, and averaged across the consecutive sections. The SNR and CNR were calculated by the following formula:

$${\text{SNR }}_{\mathrm{the}\,\mathrm{involved}\,\mathrm{paracervical}\,\mathrm{vein}}={\text{HU }}_{\mathrm{the}\,\mathrm{involved}\,\mathrm{paracervical}\,\mathrm{vein}}/{\text{SD }}_{\text{fat}}$$

$$\text{CNR}=\left({\text{HU }}_{\mathrm{the}\,\mathrm{involved}\,\mathrm{paracervical}\,\mathrm{vein}}\,-{\text{HU}}_{\mathrm{gluteal}\,\mathrm{muscle}}\right)/{\text{SD}}_{\text{fat}}$$

To investigate the diagnostic value of quantitative dual-energy CT parameters for this entity, ROIs were placed in the intravenous and intra-atrial lesions, respectively. The iodine concentration derived from ID maps and the effective atomic number on Zeff maps were measured. The energy spectrum curves were plotted with keV (range = 40–190 keV) as the abscissa and the CT value as the ordinate. The slope of the energy spectrum curve (λ_HU_) was calculated as follows: λ_HU_ = (HU_40 keV_ − HU_70 keV_)/(70 − 40), where HU_40 keV_ and HU_70 keV_ refers to the CT values at each energy level (12).

## Results

### Clinical characteristics

A 40-year-old woman presented to the intervention department complaining of edema in both lower limbs for 10 days. Physical examination revealed a depression in the lower limb edema after finger pressure was applied to the area. Additionally, she complained of a 1-year history of menorrhagia. Laboratory blood tests revealed a Tumor Abnormal Protein (TAP) level of 203.375 μm^2^; however, alpha fetoprotein (AFP), carcinoembryonic antigen (CEA), cancer antigen 19-9 (CA19-9), cancer antigen 125 (CA125), and cancer antigen 15-3 (CA15-3) levels were all within the normal range.

### Imaging findings

#### Ultrasonography features

Cardiac US imaging demonstrated a 74 mm × 34 mm solid mass within the right atrium that moved back and forth between the right atrium and right ventricle with the cardiac cycle. The mass extended down to the inferior vena cava in a long strip that floated slightly in the bloodstream. Pelvic US revealed a hypoechoic mass measuring 47 mm × 22 mm in the right posterior part of the uterus, which was closely connected with the right lateral wall of the uterus; a mixed mass measuring 52 mm × 41 mm was observed in the right rear of the uterus, which had an irregular shape, unclear boundaries, and a tortuous distal end. The two masses were partially connected. Color doppler flow imaging (CDFI) showed a blood flow signal in these two masses.

#### Computed tomography features

**FIGURE 1:**
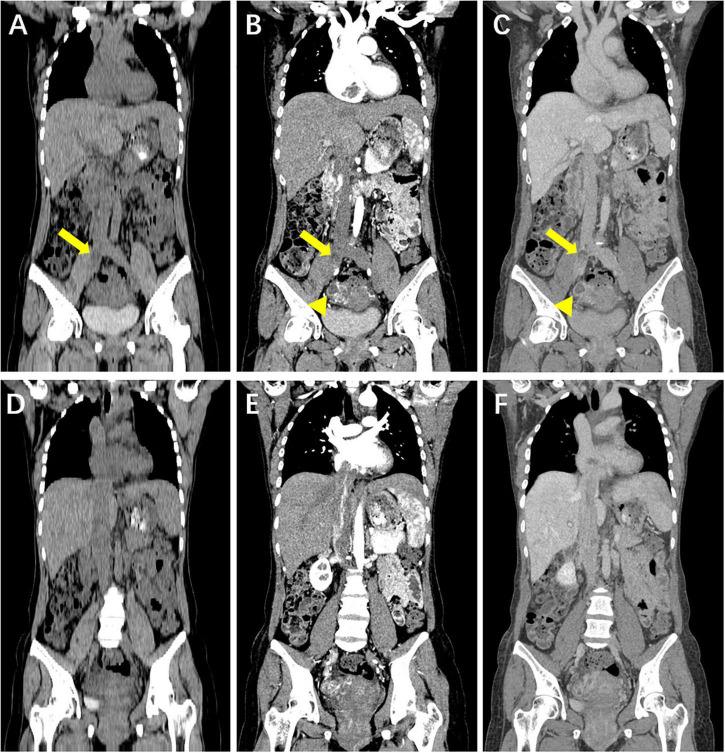
Coronal reformatted computed tomography (CT) image of the chest, abdomen and pelvis. **(A–F)** Hypoattenuating filling defect located within the right iliac veins across the inferior vena cava (IVC) into the right atrium. Contrast-enhanced CT images show that intravenous lesions demonstrated mainly moderate progressive enhancement, with localized non-enhancing necrotic areas (arrow). A circular enhanced thick-walled cystic lesion at the right of the uterine body is incidentally noted (arrowhead). **(A,D)** Unenhanced phase image. **(B,E)** Arterial phase of contrast enhancement image. **(C,F)** Portal phase of contrast enhancement image.

**FIGURE 2:**
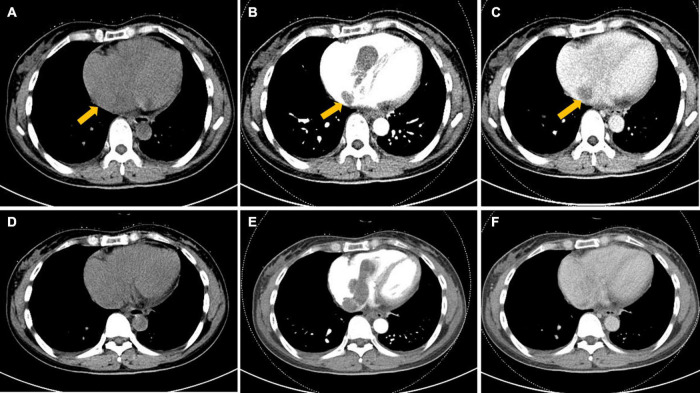
Axial computed tomography (CT) image of the heart. **(A–F)** Hypoattenuating filling defect located within the right atrium across the tricuspid valve into the right ventricle. Intracardiac lesions demonstrated mainly moderate progressive enhancement, with localized non-enhancing necrotic areas (arrow) on contrast-enhanced CT. **(A,D)** Unenhanced phase image. **(B,E)** Arterial phase of contrast enhancement image. **(C,F)** Portal phase of contrast enhancement image.

Computed tomography of the chest, abdomen, and pelvis depicted a filling defect within the tortuous and enlarged paracervical vein that extended superiorly through the right common iliac vein into the right atrium and right ventricle. The filling defect showed heterogeneous hypoattenuation, mainly progressive enhancement, accompanied by a non-enhanced low attenuation area. In addition, a circular enhanced thick-walled cystic lesion could be seen on the right of the uterine body (Figures 1, 2).

#### Magnetic resonance imaging features

**FIGURE 3:**
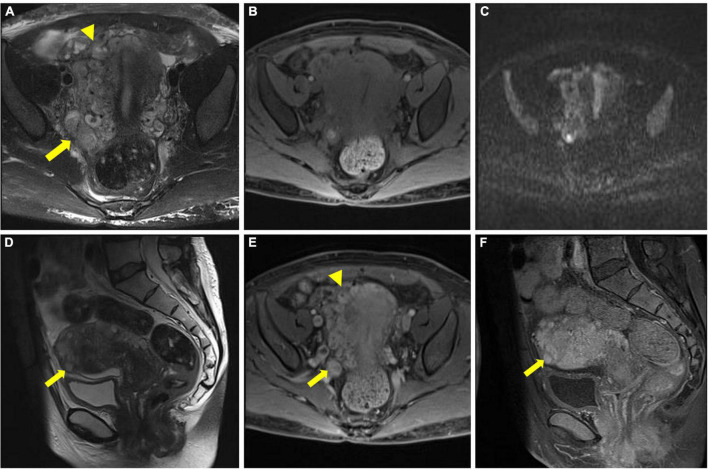
Pelvic magnetic resonance imaging (MRI) image. **(A–C)** Multiple tortuous and dilated vascular structures at the fundus of the uterus (arrowhead) and the parametrium (arrow). **(D)** Multiple nodules of varying sizes were observed at the fundus of the uterus (arrow). **(E,F)** After gadolinium-based contrast material injection, progressively enhanced lesions were noted within the lumen (arrowhead and arrow in **E**), and the nodules also presented progressive enhancement (arrow in **F**).

To further clarify the condition of the uterus, pelvic MR imaging was performed and revealed the following: ➀ Multiple nodules of varying sizes were observed at the fundus of the uterus, which showed low signals on T1-weighted images, high signals on T2-weighted images and a low signal intensity in DWI images. The nodules demonstrated progressive enhancement following intravenous administration of gadolinium-based contrast material. ➁ Multiple tortuous and dilated vascular structures with heterogeneous signal intensity were observed at the fundus and the parametrium. The vascular structure demonstrated mixed equal or high signals on T1-weighted images and mixed low or high signals on T2-weighted images. After the injection of gadolinium-based contrast material, there were progressively enhanced lesions in the lumen, which were the invaded vein. No evidence of lymph node metastasis was detected in the pelvic cavity (Figure 3).

### Subjective and objective dual-energy computed tomography image analysis

**FIGURE 4:**
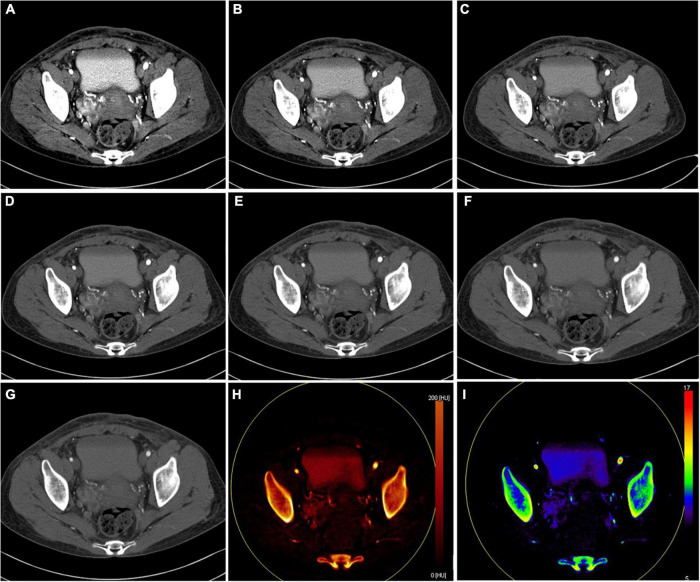
Axial dual-energy computed tomography (CT) images show tortuous and enlarged paracervical veins. **(A–G)** 40–100 keV VMIs (10-keV interval). **(H)** Iodine density map. **(I)** Z effective map.

All reconstructed dual-energy datasets are presented in Figure 4. For visualization of the involved paracervical vein, the lesion conspicuity scores were highest at 40 keV VMI. Moreover, the scores of 40–60 keV VMI, ID, and Zeff reconstructions were superior to 70 keV VMI.

**FIGURE 5:**
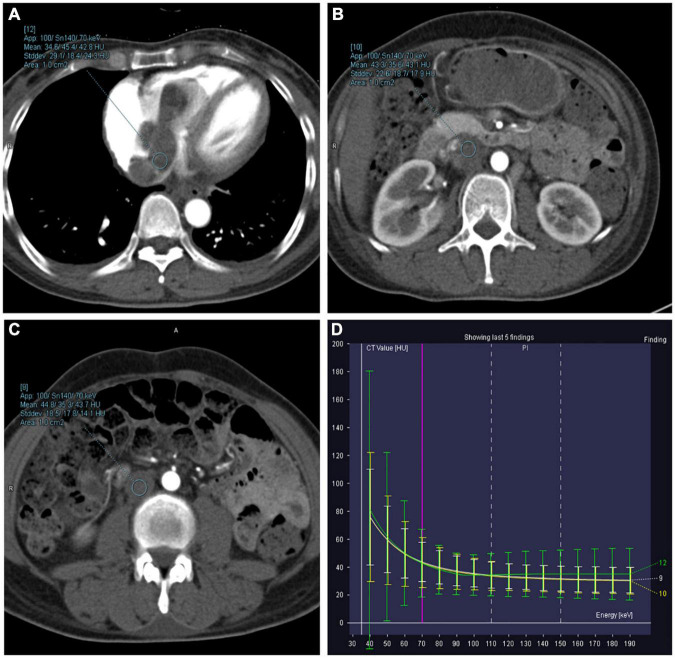
Right atrium, inferior vena cava (IVC) at the renal venous level, and IVC at the inferior mesenteric artery level-derived energy spectrum curve. The curves of each color represent the energy spectrum curves of each ROI. **(A)** Green: right atrial lesions. **(B)** Yellow: lesions of the IVC at the renal venous level. **(C)** White: lesions of the IVC at the inferior mesenteric artery level). **(D)** The energy spectrum curves represent the CT values of the ROI under different keV conditions.

The objective quantitative SNR and CNR values are displayed in Table 2. VMI, at 40 keV, exhibited the highest SNR and CNR (SNR, 7.09; CNR, 4.62). The comparison of quantitative DECT parameters between inferior vena cava and right atrial mass is presented in Table 3. Their energy spectrum curves both showed a downward trend in the range of 40–100 keV and a stable trend in the range of 100–190 keV (Figure 5). Furthermore, the corresponding slopes of IVC at the renal venous level and IVC at the inferior mesenteric artery level were almost identical (1.09 VS 1.07).

**TABLE 2:** VMI: quantitative values for CNR and SNR.

	40 keV	50 keV	60 keV	70 keV	80 keV	90 keV	100 keV
**CNR**	4.62	3.88	2.82	2.46	1.90	1.42	1.05
**SNR**	7.09	6.56	5.40	5.49	5.07	4.69	4.39

CNR, contrast-to-noise ratio; keV, kiloelectron volt; VMI, virtual monoenergetic image; SNR, signal-to-noise ratio.

**TABLE 3:** Quantitative dual-energy computed tomography (DECT) parameters between inferior vena cava and right atrial mass.

Quantitative parameters	Right atrium	IVC at the renal venous level	IVC at the inferior mesenteric artery level
IC (mg/ml)	0.8	0.7	0.6
λHU (HU/keV)	1.27	1.09	1.07
Zeff	7.84	7.79	7.7

HU, Hounsfield unit; IC, iodine concentration; Zeff, effective atomic number; λHU, slope of the spectral HU curve.

### Gross pathological features and follow-up

Single-stage operation was performed, including total hysterectomy, and resection of intracardiac, IVC, and right common iliac vein tumor. During surgical exploration, space-occupying lesions originating from the inferior vena cava could be seen in the right atrium, and the head of this entity was found to enter the right ventricle through the tricuspid valve. In addition, a long and continuous mass was observed in the inferior vena cava, blocking it and extending down to the level of the right common iliac vein. Uterine enlargement was found, which was similar to that commonly observed in women after more than 40 days of pregnancy; and there was an enlarged mass on the right side of the cervix, compressing the cervix toward the left, and tumor thrombus could be palpated in the parametrial vein. No obvious abnormality was found in the bilateral adnexa.

**FIGURE 6:**
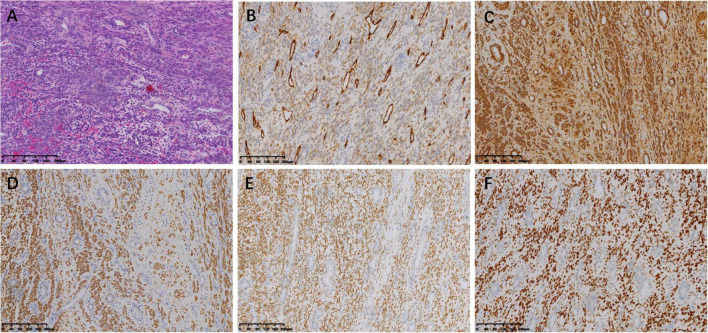
Microscopic examination of intracardiac and intravenous mass. (magnification, ×100). **(A)** Hematoxylin-eosin (HE) staining of the tumor cells. Immunohistochemical staining for CD10 **(B)**, SMA **(C)**, Desmin **(D)**, ER **(E)**, and PR **(F)**.

**FIGURE 7:**
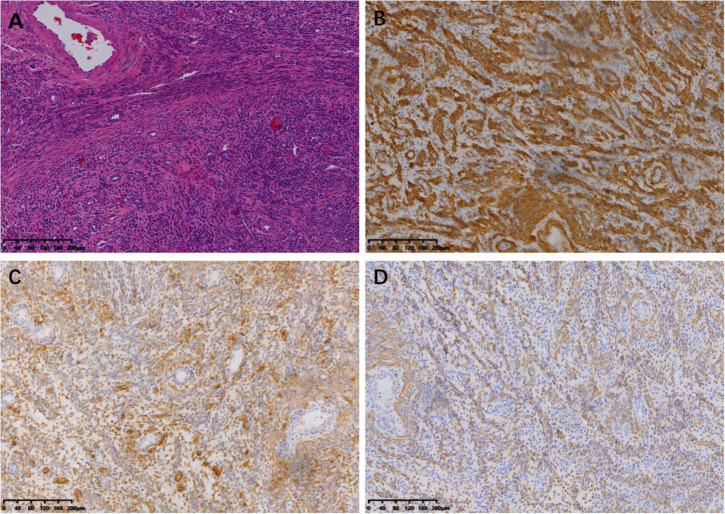
Microscopic examination of uterine lesion (magnification, ×100). **(A)** HE staining of the tumor cells. Immunohistochemical staining for SMA **(B)**, CD10 **(C)**, and Desmin **(D)**.

The tumor was predominantly composed of spindle cells that were intertwined with each other. These cells had relatively abundant eosinophilic cytoplasm and oval or elongated nuclei with coarse-grained chromatin structure. Mitotic figures were rarely seen. Focal necrosis of the tumor cells located in the heart was observed, whereas no definite necrosis was observed in the uterus. Regarding immunohistochemistry, the tumor cells of the uterine mass were positively immunolabeled with smooth muscle actin (SMA), desmin, and CD10. The labeling index of Ki-67 index was less than 10%. The immunostaining results of the tumor cells in the venoatrial mass were the same as those of the cells in the uterine mass. In addition, estrogen receptor (ER) and progesterone receptor (PR) staining were performed to determine whether the venoatrial mass originated from the uterus, and the results showed that the labeling index of ER and PR was more than 80 and 90%, respectively (Figures 6, 7).

The final pathological diagnosis was mixed endometrial stromal and smooth muscle tumor of the uterus with inferior vena cava metastasis into the right atrium.

The patient recovered uneventfully and was followed up by CT for 8 years without any complications or recurrence.

## Discussion

Mixed endometrial stromal and smooth muscle tumor is a rare and miscellaneous mesenchymal uterine tumor characterized by the presence of endometrial stromal and smooth muscle tissues (each component > 30%) (13). The biological behavior of these mixed tumors depends on the endometrial stromal tumor; however, the differentiation of the two components is difficult due to their overlapping pathologic features (14). MESSMT has the potential for vascular invasion and mimics intravenous leiomyomatosis (IVL) grossly.

Compared with that of uterine stromal tumors and smooth muscle uterine tumors, the incidence of MESSMT is very low, especially with intracardiac extension. MESSMT was first reported by Tang under the name of endometrial stromal tumor in 1979 (1), while the intracardiac extension of this entity was not proposed until 1987 by Whitlash et al. (2). Incredibly, to date, only five reports on MESSMT with intracardiac extension have been published in PubMed. The age range of the MESSMT patients is 29–68 years (6); however, MESSMT with intracardiac extension typically appears in patients ranging in age from 24 to 50 years. In the current study, the patient was 40 years old, which is close to the average age of the five previously reported patients (42.2 years). The clinical presentation of MESSMT is atypical, and an abdominal mass or enlarged uterus are the most common signs (6). Abnormal bleeding such as vaginal spotting after menopause (14), and hypermenorrhea, as in the present case, can also be present. As tumors invade the iliac veins, IVC, right atrium, and right ventricle, patients can experience edema of the inferior limbs, Budd-Chiari syndrome, tachycardia and arrhythmias, dyspnea, cardiac tamponade and even sudden death (13). Notably, the majority of patients who were diagnosed with MESSMT with intracardiac extension had a history of surgery for uterine leiomyoma, which is similar to that reported for intravenous leiomyomatosis (15). Tumor markers of MESSMT with intracardiac extension have been rarely reported, although the patient in the case reported by Huang et al. had a slight increase in CA125 (6). In the present study, the only abnormally elevated tumor marker was TAP, which persisted for 7 years.

Careful preoperative imaging examination is of great clinical significance for planning the treatment of pelvic tumors with intravenous extension (5). Since this disease includes both uterine lesions and extrauterine lesions, we intend to explore the diagnostic value of different imaging tools for these two types. Ultrasonography is the most frequently used, economical, and simple imaging means for the assessment of the female reproductive system (16). The US characteristics of the uterus in patients with MESSMT with intracardiac extension have not been previously described in detail. In our study, we found that the uterine volume of the patient increased, and a mixed mass with an irregular shape could be seen in the right posterior part of the uterus. The boundary was unclear, and the distal end route was tortuous. CDFI showed a blood flow signal in the mass. Nevertheless, the diagnostic value of ultrasound for tumors is extremely limited, especially for this complex mixed tumor. For pelvis imaging, MRI has excellent soft tissue contrast resolution and allows for direct multiplanar imaging and offers variable imaging parameters to aid in tumor diagnosis (17). Existing reports of MESSMT with intracardiac extension only describe a large mass in the abdominal cavity on MRI. However, in the present study, multiple nodules of different sizes were noted in the fundus of the uterus, showing progressive enhancement, which may be the primary lesions of the tumor. In addition, dilated and tortuous veins could be seen in the right wall of the uterus and right parametrium, and this area showed progressive enhancement, with intravascular lesions of mild progressive enhancement. For extrauterine lesions, CT may be more suitable than ultrasound or MRI to define and show the full extent of tumor expansion due to their high spatial resolution and high-speed imaging ability (18, 19). Huang et al. and Zhang et al. reported patients with MESSMT with intracardiac extension, and CT images showed an intravascular filling defect within the right common iliac vein or pelvic veins, extending through the IVC into the right atrium or right ventricle (5, 6). Furthermore, Coganow et al. not only observed an enhanced mass from the right common iliac vein invading the right ventricle but also described that the mass passed through the main pulmonary artery to the right pulmonary artery and even into the right lobar pulmonary arteries on CT (4). In our case, enhanced CT showed a hypoattenuating filling defect within the paracervical vein extending superiorly through the right common iliac vein and IVC into the right atrium to the ventricle, and the involved vessels were markedly expanded. After contrast medium injection, the filling defects demonstrated mainly moderate progressive enhancement, with localized non-enhancing necrotic areas. Therefore, our case is characterized by a small uterine primary lesion with extensive vascular extension.

From a clinical point of view, it is difficult to differentiate MESSMT with intracardiac extension from IVL. Moreover, tomographic differential diagnosis of the two is even more challenging. On CT imaging, IVL usually manifests as hypoattenuating intravascular filling defects and associated uterine leiomyomas, with heterogeneous enhancement and, occasionally, blood vessels in the lesion (20). Meanwhile, pelvic and intravascular masses are continuous. Tumor extension is predominantly unilateral, often involving the uterine vein (which extends to the internal iliac vein, common iliac vein, and IVC) (21). Of note, although rare, these tumors can also extend via the ovarian venin route (with subsequent extension to the renal vein and IVC) (22). On MRI, the findings of the intravascular tumor typically show a mass of distended and folded tubular structures and a “sausage-like” appearance, with a mildly to significantly high-intensity signal on T2-weighted images and an iso- to mildly high-intensity signal on T1-weighted images. The tumor may have a heterogeneous signal intensity with homogeneous or heterogeneous enhancement (21). Hence, differential diagnosis through tomographic imaging of MESSMT with intracardiac extension with IVL is difficult because both are uterine tumors growing along the vein and have overlapping imaging features. In summary, the diagnostic confirmation of MESSMT with intracardiac extension relies on comprehensive clinical, imaging, and histopathological evidence.

Dual-energy computed tomography is a landmark CT acquisition method that collects two different energy spectra as input for material decomposition algorithms, and it can offer clinically informative image sets (23). Virtual monoenergetic images derived from DECT have been shown to improve soft tissue contrast and increase the conspicuity of solid organ lesions (24). Color-coded iodine density and Z effective reconstruction images have also been proven to perform well in the subjective assessment of lesions since the human eye is more sensitive to color than to grayscale (25). Regarding the visualization effect of the dual-energy dataset on the early vascular invasion of MESSMT, we found that lesion visualization with 40 keV reconstruction images is better than other VMIs, while color-coded ID and Zeff reconstructed images also achieve satisfactory performance. This finding is similar to previous research results (8, 9, 25). Based on objective assessment of the image quality, higher CNR corresponds to lower keV because higher CT attenuation of the vein lesions leads to higher contrast to the background. Thus, these dual-energy maps may contribute to the early detection of MESSMT with intravascular extension. In our study, quantitative DECT parameters and energy spectrum curve of the atrial mass were very close to those of intravenous mass. This finding may be attributed to the fact that dual-energy material composition allows for the evaluation of tumor homology.

The most effective treatment is dependent on complete surgical resection, and multidisciplinary combined surgery is necessary due to the complexity of the tumor (5). Staged surgery approaches are recommended for some patients, such as those with a poor clinical condition who are not candidates for prolonged surgery or those with extensive lesion invasion, which cannot be completely removed in a one-stage procedure (6). Regarding the surgical strategy for MESSMT with intracardiac extension, a thoracic approach is needed to remove the intracardiac portion of the tumor, and an abdominal pelvic approach is needed to remove the intracaval and pelvic portions (2–6). Of note, similar to the surgical strategy of IVL, the abdominal surgical approach is recommended because tumor attachment to the IVC prevents complete extraction of the tumor via the right atrium, which could lead to caval injury and retroperitoneal hemorrhage (21). Moreover, in the pelvic region, hysterectomy and single or bilateral salpingo-oophorectomy were performed in most cases (2–6). Of the five patients previously reported, two demonstrated evidence of recurrence 1 and 10 years after the operation (2, 3). One of the patients who experienced recurrence died of cardiovascular effects caused by intravascular growth of the tumor (2). Thus, long-term and regular surveillance after surgery is necessary. Follow-up imaging may include US, CT, and MRI evaluation of the uterine bed, IVC and iliac veins. In addition, the research indicates that antiestrogen therapy may reduce the possibility of recurrence (3).

## Conclusion

Although MESSMT with intracardiac extension is exceedingly unusual, it is a lesion with malignant biological behavior that can be lethal and is prone to recurrence. Imaging examination can characterize the primary lesion of the uterus and provide clarity regarding the range of the intravascular extension of the tumor, which is closely related to the formulation of the surgical scheme. Nevertheless, the imaging appearance of MESSMT with intracardiac extension overlaps highly with IVL, so the final diagnosis requires further pathological and immunohistochemical evidence. Complete surgical resection and close long-term follow-up are necessary for these patients. Concurrently, this study also shows that 40 keV VMIs are most helpful to improve the visualization of paracervical abnormal vascular changes for the early detection of MESSMT with intravascular extension. Quantitative DECT parameters and energy spectrum curves can provide support for the determination of the extensive lesion homology.

## Data availability statement

The original contributions presented in this study are included in the article/supplementary material, further inquiries can be directed to the corresponding author.

## Ethics statement

This study was reviewed and approved by the Medical Ethical Committee of Zhengzhou University. The requirement for informed consent was waived.

## Author contributions

Y-yL: manuscript preparation, literature research, and data analysis. ZY: manuscript preparation and literature research. RW: manuscript review and data collection. K-sC: guidance of pathological knowledge. S-wY, JL, and X-mG: guidance of imaging knowledge. C-mD: imaging data collection and analysis. Z-xW: language editing. XZ: dual-energy knowledge guidance. J-bG: study conception and design, manuscript review, and guarantor of integrity of the entire study. All authors contributed to the article and approved the submitted version.
